# Supplementary figures and images for: A Combinatorial Q-Locus and Tubulin-Based Polymorphism (TBP) Approach Helps in Discriminating Triticum Species
Source: Genes (Basel). 2022 Apr 1;13(4):633. doi: 10.3390/genes13040633 (PMC9029001; doi:10.3390/genes13040633)

Figure S1. CE-TBP specific peaks disseminate spelt cultivars from common wheats.

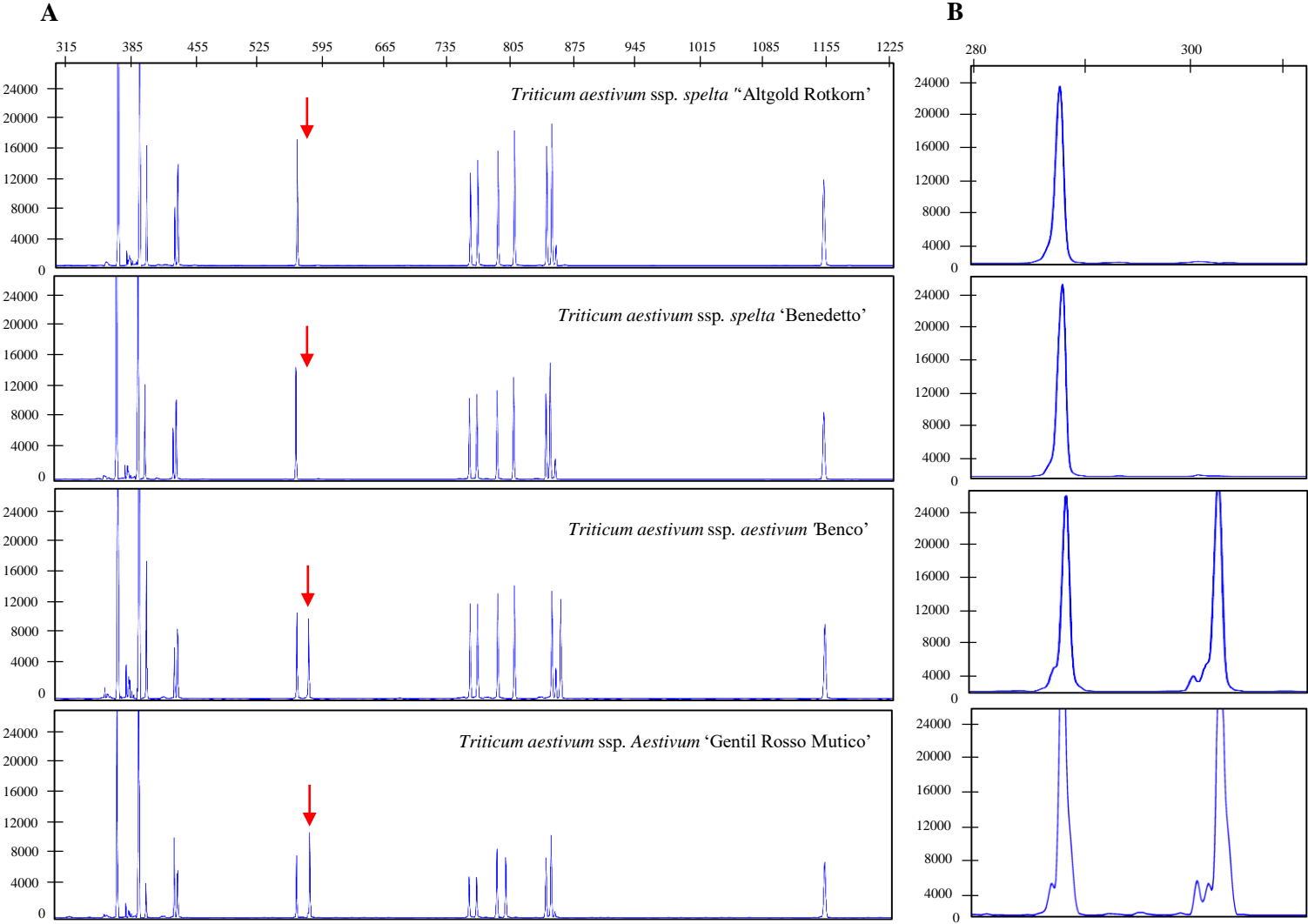

Supplement: Supplementary file 1 [file genes-13-00633-s001.zip › genes-1638769 - supplementary Figure S1.pdf]
